# A challenging diagnosis: The overlap of hepatorenal polycystic disease and amebiasis

**DOI:** 10.1016/j.idcr.2026.e02511

**Published:** 2026-02-06

**Authors:** Marie Jumpertz, Coralie L’Ollivier, Estelle Menu

**Affiliations:** a Assistance publique-hôpitaux de Marseille (AP-HM), Service d’infectiologie, Marseille, France; b Aix Marseille Univ, SSA, APHM, RITMES, Marseille, France; c Assistance publique-hôpitaux de Marseille (AP-HM), Service de Parasitologie-Mycologie, Marseille, France

**Keywords:** *Entamoeba histolytica*, Amoebiasis, Liver abscess, Polycystic kidney disease

## Abstract

A 71-year-old retired man returning from Senegal was found to have multiple hepatorenal cysts associated with a colonic mass on CT. Initially misdiagnosed, stool and abscess puncture analyses revealed intestinal and extraintestinal amebiasis. On the other hand, genetic testing suggested hepatorenal polycystic disease, demonstrating the coexistence of these two pathologies.

## Highlights

• Genetic hepatorenal polycystosis and hepatic amoebiasis can co-exist.
• Amoebic liver abscess must be considered in travelers with hepatorenal polycystosis.
• The use of corticosteroids can have devastating effects on patients with amoebiasis.
• Before using corticosteroids, screen a traveller with colitis for amoebic dysentery.

## Introduction

Amoebiasis is caused by an anaerobic protozoan parasite *Entamoeba histolytica*. Every year, nearly 50 million people worldwide contract this infection, primarily in developing countries [1]. In developed countries, this infection therefore mainly affects travelers. Clinical manifestations vary widely, ranging from asymptomatic to severe disease. Intestinal amebiasis manifests with nonspecific symptoms such as cramping abdominal pain, bloody diarrhea, and weight loss without fever [2]. If intestinal amoebiasis is not treated, it can progress to invasive extra intestinal forms among which amoebic liver abscess is the most common.

In industrialized countries where the prevalence is low, amoebiasis can be misdiagnosed. When it appears as an amoeboma, it can lead to misdiagnosis as colon cancer depending on their location [3]. Similarly, amoebic colitis is a great mimicker of inflammatory bowel diseases [4]. When faced with liver lesions, several causes can be considered: acquired or congenital, but in some cases, both forms may coexist, requiring careful attention [5].

We reported a case of an elderly man returning from Senegal presenting colitis and multiple hepatorenal cysts initially misdiagnosed. It highlights the challenge that managing amoebiasis outside endemic areas can represent, particularly when multiple pathologies overlap.

## Case presentation

A 71-year-old retired man who travels regularly to Senegal was referred to our department by his gastroentreologist for imaging findings of colonic mass of undetermined origin.

The patient's medical history began with a consultation with his general practitioner for abdominal pain associated with mucus-bloody stools. After a unique negative parasitological stool examination, the doctor referred the patient to a gastroenterology consultation. A colonoscopy was performed, showing infiltration of the cecum and rectum with a mass measuring 105x89x66 mm, raising the possibility of inflammatory bowel disease or lymphoma. The biopsy led to a diagnosis of a neutrophilic colitis. A treatment with corticosteroids was then initiated at a dose of 60 mg/day for 7 days. This treatment was unsuccessful, and four days after discontinuation, the patient developed a fever of 39°C.

**Fig. 1:**
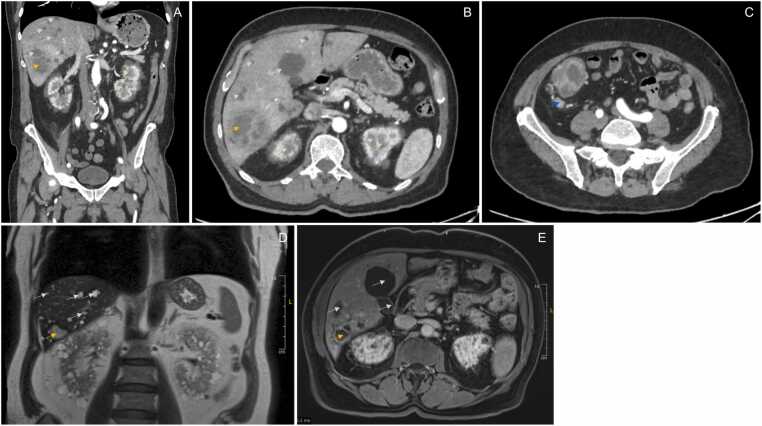
CT scan upon arrival (A) Frontal section after injection of iodinated contrast medium in the arterial phase showing innumerable hepatic and renal cysts (a few represented with green arrow) with poorly defined lesion formation at the hepatic apex (yellow arrow); (B) Axial section after injection of iodinated contrast medium in the arterial phase focusing on the hepatic amoebiasis abscess (yellow arrow); (C) Axial section after injection of iodinated contrast medium in the arterial phase showing the caecal pseudo-mass (blue arrow). MRI after 3-months follow-up (D) Frontal section showing innumerable hepatic and renal cysts (a few represented with green arrow) with hepatic abscess of 2,3 cm with T2-weighted intermediate signal (yellow arrow); (E) Axial section showing cocarde contrast of the hepatic abscess after gadolinium injection (yellow arrow)

Upon arrival in the infectious diseases department, the patient was asthenic, febrile, dehydrated, and reported weight loss of approximately 10 kg over the past month. An abdominal-pelvic CT scan subsequently revealed a focal colitis in the cecum and rectum, more suggestive of an infectious cause, as well as hepatorenal polycystosis with a hepatic abscess of 5.5 cm (Fig. 1A, Fig. 1B, Fig. 1C).

Parasitological examination of stools showed at the point the presence of *Entamoeba histolytica* cysts by direct examination, confirmed by specific qPCR targeting the hemolisin gene [6]. As stated in the literature, a single stool examination can return negative due to the inconsistent excretion of the parasite. It is essential to perform repeated stool examinations [7]. Amebiasis serology was positive by *Entamoeba histolytica* IgG ELISA (BORDIER Affinity Products, Crissier, Suisse), at index 2.31 (normal value < 0.9) and haemagglutination ELI.H.A *Amoeba* (ELITechGroup SAS, Puteaux, France), at titer 1/1280 (normal value < 1/80).

A treatment with metronidazole 500 mg, three times a day, was started for 10 days, followed by paromomycin 500 mg, three times a day for 7 days. An improvement in diarrhoea was seen within 1 day of starting treatment. The 3-months follow-up hepatic MRI showed an appearance compatible with a hepatic cystic infection in segment VI measuring 2.3 cm with cocardial contrast after gadolinium injection, and the disappearance of the colonic mass (Fig. 1D, Fig. 1E). A decrease of the serology was observed (*E. histolytica* IgG ELISA index 1.78 and haemagglutination ELI.H.A titer 1/640). At this time, the abscess puncture was positive for *E. histolytica* qPCR. A CT scan performed at 6 months showed regression of the abscess.

Genetic testing revealed a mutation consistent with autosomal dominant polycystic kidney disease.

## Discussion

Amebiasis can lead to intestinal and extra-intestinal disease with a wide range of symptoms, from the asymptomatic form to the lethal form [8], [9]. Since the symptoms are nonspecific and due to the low prevalence of amoebiasis in industrialized countries, diagnosis can be challenging. Cecal amebiasis can mimic inflammatory bowel disease leading to the use of corticosteroids which may worsen the patient's general condition and lead to death [4], [10]. It is important not to rush into immunosuppressive treatment before formally ruling out amoebiasis in a traveler. In our case, the patient rapidly deteriorated after starting corticosteroid therapy, with a decline in his general condition and the onset of fever. It is therefore essential to screen for amoebic dysentery in a traveler presenting with colitis, particularly before the initiation of corticosteroid therapy [4], [8].

In our case, the initial amoeba test, which was based solely on direct stool testing, was negative. Unique direct examination of stool sample has low sensitivity due to intermittent shedding and could lead to misdiagnosis [2]. It is better to conduct at least three stool parasitological exams. Recommendations now include systematic screening for *Entamoeba histolytica* using specific PCR, combined with a panel of other parasitic diseases in addition to direct examination as part of a syndromic approach [11]. Ideally, testing for anti-amebic antibody in serum should also be performed [2]. This was done as soon as the patient arrived in our department and led to a successful diagnosis. Syndromic approach could have prevented an initial misdiagnosis.

The particularity of our case also lies in its overlap with polycystic hepatorenal disease, which may have caused confusion and masked the abscess. There are few reported cases of diagnostic difficulty due to the concomitant presence of hepatorenal polycystosis and hepatic amebiasis [12], [13], [14]. Amoebiasis is a neglected cause of liver abscess in travelers and should not be overlooked in any case of liver abscess [15].

## Conclusion

In returning travelers with colitis, amoebiasis should be considered and screened for before initiating immunosuppressive therapy. The parasitological workup should include repeated parasitological exams, and a specific PCR screening as part of a syndromic approach. It is important to keep in mind that conditions such as amoebic abscess and hepatorenal polycystic disease can overlap.

## CRediT authorship contribution statement

**Estelle Menu:** Writing – review & editing, Supervision, Investigation. **Marie Jumpertz:** Writing – review & editing, Writing – original draft, Investigation. **Coralie L’Ollivier:** Investigation.

## Consent from patient

Written consent of patient had been obtained. The study has been registered on the AP-HM Health Data Access Portal under the number PADS 24–308.

## Funding

There are no sources of funding.

## Declaration of Competing Interest

The authors declare that they have no known competing financial interests or personal relationships that could have appeared to influence the work reported in this paper.
